# A Double-blind Randomized Trial of Oral Chlorhexidine Gluconate for Treatment of Oral *Staphylococcus aureus* Colonization in Healthy Children

**DOI:** 10.1093/ofid/ofag072

**Published:** 2026-02-18

**Authors:** Lucia Liu, Bryn Launer, Evelyn Flores, Greg Tchakalian, Barry Kreiswirth, Michael Bolaris, Tae Kim, Kelly D Young, Loren G Miller

**Affiliations:** Duke University School of Medicine, Durham, North Carolina, USA; Vanderbilt University Medical Center, Nashville, Tennessee, USA; Lundquist Institute for Biomedical Innovation at Harbor-UCLA Medical Center, Torrance, California, USA; University of Southern California School of Pharmacy, Los Angeles, California, USA; Center for Discovery and Innovation, Hackensack, New Jersey, USA; Lundquist Institute for Biomedical Innovation at Harbor-UCLA Medical Center, Torrance, California, USA; Rancho Los Amigos Medical Center, Downey, California, USA; Lundquist Institute for Biomedical Innovation at Harbor-UCLA Medical Center, Torrance, California, USA; Lundquist Institute for Biomedical Innovation at Harbor-UCLA Medical Center, Torrance, California, USA; David Geffen School of Medicine at UCLA, University of California, Los Angeles, California, USA; Departments of Pediatrics and Emergency Medicine, Harbor-UCLA Medical Center, Torrance, California, USA; Lundquist Institute for Biomedical Innovation at Harbor-UCLA Medical Center, Torrance, California, USA; David Geffen School of Medicine at UCLA, University of California, Los Angeles, California, USA; Division of Infectious Diseases, Harbor-UCLA Medical Center, Torrance, California, USA

**Keywords:** chlorhexidine gluconate, decolonization, oropharyngeal colonization, randomized clinical trial, *Staphylococcus aureus*

## Abstract

**Background:**

*Staphylococcus aureus* is the most common cause of skin and soft tissue infection (SSTI). Nasal *S. aureus* colonization may precede SSTI, and decolonization may decrease SSTI risk. However, many *S. aureus–*colonized persons are oropharyngeally colonized, sometimes without concomitant nasopharyngeal colonization. However, there are few data on oropharyngeal *S. aureus* decolonization, especially in children.

**Methods:**

We performed a prospective, double-blind, randomized controlled clinical trial of twice-daily 0.12% chlorhexidine gluconate (CHG) gargle vs a placebo for 7 days in healthy children oropharyngeally colonized with *S. aureus*. At each study visit (days 1, 8, 29), throat and nares cultures were performed. All *S. aureus* isolates underwent *spa* typing.

**Results:**

We screened 189 children, 120 (63%) of whom had *S. aureus* oropharyngeal colonization; of these, 67 (56%) were randomized. The median participant age was 11 years (mean, 11.7), and 27 (40%) were female. In the intention-to-treat population, oropharyngeal colonization at day 8 was 45% (15/33) and 79% (27/34) in the CHG and placebo groups, respectively (*P* = .004), and 61% (20/33) vs 85% (29/34) at day 29 (*P* = .03). Among children who were oropharyngeally decolonized at day 8 but positive for *S. aureus* at day 29, 8/12 (66%) exhibited a new *spa* type compared with baseline.

**Conclusions:**

We found that a 7-day 0.12% chlorhexidine gluconate mouthwash regimen significantly reduced *S. aureus* oropharyngeal colonization compared with placebo. This difference persisted at day 29, suggesting that CHG mouthwash may be a promising adjunctive decolonization agent that may decrease the high SSTI recurrence risk in children.

## Teaser

In this double-blind, randomized trial, chlorhexidine gluconate (CHG) mouthwash significantly reduced oropharyngeal Staphylococcus aureus colonization in children compared to placebo through 29-day follow-up. CHG shows potential as an adjunct to prevent recurrent skin and soft tissue infections in children.

Skin and soft tissue infections in children account for >2 million visits to primary care and emergency departments annually in the United States [1]. The most common cause of skin and soft tissue infections (SSTIs) is *Staphylococcus aureus*, with methicillin-resistant *S. aureus* (MRSA) accounting for over one-third of cases in pediatric settings [2–4]. SSTIs have an annual incidence of ∼4 in 100 children [5]. Furthermore, recurrences of *S. aureus* SSTI in children are common, and reported rates of recurrence are 20%–70%, with particularly high rates among those with MRSA SSTIs [6]. Even after systemic antibiotic therapy for an SSTI, *S. aureus* colonization persists in nearly one-half of patients [7]. There are data suggesting that colonization plays an important role in SSTI recurrence [8–12].

Studies investigating the utility of skin and/or nasal decolonization methods in preventing recurrence of *S. aureus* SSTIs have demonstrated mixed success. In children and their household contacts, 1 study showed that chlorhexidine baths, intranasal mupirocin, and environmental decolonization lowered SSTI recurrence among participants with prior-year infections [13]. In adults, a 1-year trial of nasal mupirocin reduced both nasal colonization and subsequent SSTI recurrence [14]. However, many studies have found that improvements in decolonization do not consistently translate into reduced SSTI rates [15–17]. For example, Fritz et al. randomized children with SSTIs to either skin decolonization with chlorhexidine (CHG) and nasal decolonization with mupirocin of the affected child or CHG skin decolonization plus mupirocin given to the whole household [6]. While decolonization of the whole household was superior (54% vs 72% SSTI recurrence rate during the 12-month follow-up period), unfortunately even in the more aggressively treated group, SSTI recurrence rates remained high. Similarly, a study using bleach baths alone to eliminate *S. aureus* colonization in children failed to reduce infection rates [18]. These results suggest that while skin and/or nasal decolonization is achievable, its effectiveness in preventing recurrent SSTIs is inconsistent. The cause of the limited efficacy is unclear, but it may in part be due to the presence of untreated *S. aureus* reservoirs.

While the most common location for *S. aureus* colonization has traditionally been thought to be the anterior nares, data have demonstrated that oropharyngeal *S. aureus* colonization is common (30%), and its prevalence approaches or exceeds that of the nasopharynx [19, 20]. In children, several studies have shown that oropharyngeal *S. aureus* colonization may persist longer than nasal colonization and occur in the absence of concurrent nasal colonization [21, 22]. Molecular analyses have shown that colonizing strains from both the nares and oropharynx tended to match invasive isolates, indicating that both sites may independently contribute to infection risk [23]. However, topical decolonization studies of the oropharynx using chlorhexidine (CHG) gargle have thus far been limited to adults [24], and although 1 study has examined the effect of systemic antibiotics on oropharyngeal colonization [25], no studies have examined the efficacy of targeted oropharyngeal decolonization in children. Therefore, we set out to investigate the efficacy and tolerability of CHG gargle for oropharyngeal *S. aureus* decolonization in children.

## METHODS

### Study Design and Population

We performed a prospective, double-blind, randomized controlled clinical trial of 0.12% CHG gargle vs a placebo saline used twice daily for 7 days for decolonization of *S. aureus* in the oropharynx of children colonized with *S. aureus*.

From May 2015 through October 2017, participants were recruited at Harbor–UCLA Medical Center in Torrance, California, from urgent care clinics, emergency departments, and affiliated clinics. Children were eligible for enrollment if they were between the ages of 5 and 18 and were able to gargle, undergo nasal and oropharyngeal swabbing, and return for follow-up visits. The lower limit of age was chosen as pilot data showed that children <5 years are unlikely to be able to gargle (unpublished data). Children were excluded if they had a known immunodeficiency, had received systemic antibiotics in the past 28 days, were likely to receive systemic antibiotics or be hospitalized in the subsequent 28 days, or had received any of the following in the past 6 months: hemodialysis, peritoneal dialysis, central venous catheter placement, or systemic chemotherapy.

Written informed consent was obtained from the parents in their preferred language (English or Spanish). Verbal assent was obtained from patients aged 7–12, and written assent was obtained from patients older than 12. The protocol was approved by the Institutional Review Board of Lundquist Institute for Biomedical Innovation at Harbor-UCLA Medical Center.

After informed consent was obtained, participants were asked to demonstrate their ability to gargle using water. Participants who could not gargle were then removed from the study. To determine if the consented participant had *S. aureus* colonization, we initially screened participants for *S. aureus* oropharyngeal colonization using a GeneXpert MRSA/SA rapid polymerase chain reaction (PCR) assay (Cepheid, Sunnyvale, CA, USA), which allowed us to obtain colonization results within 1 hour. Colonization was then confirmed for all participants as described below, and those with negative results by this method were removed from the study. The anterior nares was also swabbed and screened for *S. aureus* colonization as described below due to the potential for recolonization from the nares reservoir.

### Study Medication

Participants were randomly assigned in a 1:1 ratio in blocks of 6 to receive either 0.12% CHG mouthwash or an identically appearing and tasting placebo saline mouthwash. Dosing of the study medication was twice daily for 7 days. Each dose of mouthwash was 15 mL, as measured by a provided measuring cap. Participants were instructed to gargle the mouthwash for 30 seconds. Randomization was done by the local Investigational Drug Services pharmacy. All other research staff and participants were blinded to treatment allocation. Participants were also provided with new disposable toothbrushes to use with the mouthwash regimen in order to prevent recolonization from *S. aureus*–colonized toothbrushes.

### *Microbiologic* Studies and Demographic Data

Swab cultures from the oropharynx and the anterior nares were processed using enrichment broth culture followed by selective agar plating for *S. aureus*. Swabs were incubated overnight in a 7.0% NaCl tryptic soy broth and then plated on mannitol salt agar to select for *S. aureus* and mannitol salt agar with 25 mg/mL oxacillin sodium monohydride to select for MRSA. Plates were incubated overnight and then read. Samples positive for *S. aureus* were banked in 50% glycerol in a −80°C freezer. Participants and/or their parents were surveyed about demographic characteristics and coexisting medical conditions using a standardized survey. Participants underwent 2 subsequent visits, at the end of treatment (day 8) and 1-month follow-up (day 29), at which time anterior nares and oropharyngeal samples were again collected. Additionally, information about compliance and possible medication side effects was obtained with the use of diary and standardized surveys. All participants were compensated for their time.

All *S. aureus* isolates underwent staphylococcal protein A sequencing *(spa* typing) using methods previously described [26].

### Statistical Analysis

Our primary study outcome was eradication of *S. aureus* oropharyngeal colonization after 7 days of treatment with the mouthwash using an intention-to-treat (ITT) analysis, with the assumption that all missing data (visits) were associated with *S. aureus* colonization. The study was designed as a noninferiority trial in which we planned to achieve 85% power to detect a 40% absolute difference in eradication between groups (10% for placebo and 50% for CHG). Based on this assumption, we would need 54 participants. Enrollment was continued until 54 participants completed the study at day 29; any additional participants still in the trial at that point were allowed to continue. Secondary analysis included oropharyngeal colonization using an as-treated analysis at day 8 and oropharyngeal colonization using ITT and as-treated analyses at day 29.

Additional planned comparisons included differences in demographic and clinical characteristics between groups, differences in oropharyngeal colonization at day 29 among subgroups of treatment subdivided by colonization status at day 8, and nares colonization at days 8 and 29.

Comparisons between categorical variables were done via chi-square or Fisher exact test and between continuous variables as *t* tests, as appropriate. Statistical analyses were performed using SAS, version 9.1 (SAS Institute Inc, Cary, NC, USA).

## RESULTS

### *Demographic* and Clinical Characteristics of Participants

**Figure 1.**
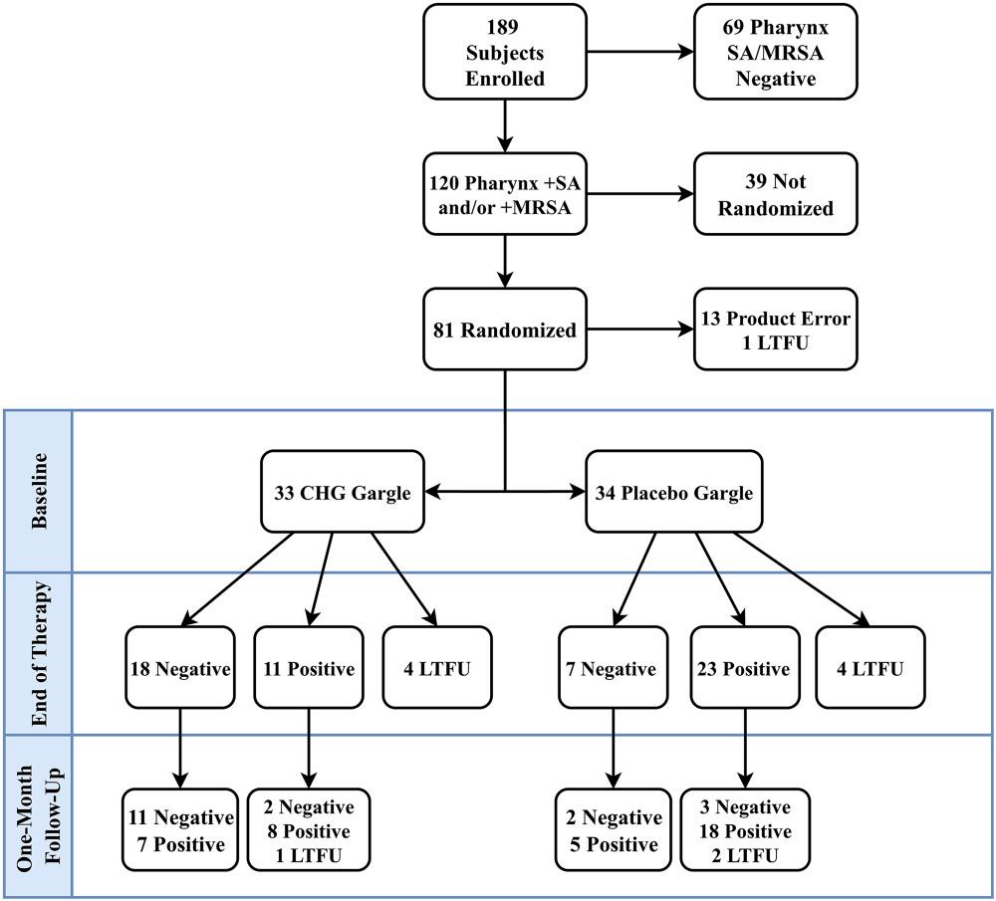
Oropharyngeal *Staphylococcus aureus* colonization across study visits. Abbreviations: LTFU, lost to follow-up; MRSA, methicillin-resistant *Staphylococcus aureus;* SA, *Staphylococcus aureus.*

A total of 189 participants were enrolled. Of these, 120 (63%) were positive for *S. aureus* oropharyngeal colonization. Of those 120 participants, 81 (68%) were randomized to receive the study drug (Figure 1). Of the 39 consented participants who were not randomized, 31 were unable to be contacted (not returning calls, incorrect numbers given) after the enrollment visit but before starting study drug, 4 withdrew from the study, 2 were not randomized due to being prescribed antibiotics upon initial screening, and 2 were excluded due to providers’ expectation of re-hospitalization (Figure 1).

After the first 13 participants were randomized, the study team had concerns that the research pharmacy was not preparing investigational product as per study protocol. The study team reviewed pharmacy procedures and corrected potential errors. Because of potential concerns with study product for the first 13 participants, these 13 participants were excluded from the analysis, leaving 67 analyzable participants. Among the analyzable participants, 33 participants received CHG, and 34 received placebo.

Among all participants, the median age (mean, range) was 11 (11.7, 5–17) years; 40 (60%) were male and 27 (40%) female, 58 (87%) were Hispanic, 4 (6%) were African American, 3 (4%) were Asian/Pacific Islander, 1 (1%) was Caucasian, and 1 (1%) was other race/ethnicity. Four (6%) had a history of skin infection, and 23 (34%) were on at least 1 oral, inhaled, or injectable concomitant medication. There were no significant demographic or clinical differences between the groups, including the frequency and major classes of concomitant medication use (Table 1). At baseline, 11/81 (13%) of the oropharyngeal *S. aureus* isolates and 5/81 (6%) of the nares isolates were MRSA.

**Table 1.** Demographics and Clinical Information Among Study Participants

…	All Participants (n = 67)	Chlorhexidine Gargle (n = 33)	Placebo Gargle (n = 34)
Age, mean, y (median, range, y)	11.7 (11, 5–17)	11.0 (10, 4–17)	12.4 (13, 5–17)
Gender	…	…	…
Female, No. (%)	27 (40)	14 (42)	13 (38)
Male, No. (%)	40 (60)	19 (58)	21 (62)
Race/ethnicity	…	…	…
Hispanic, No. (%)	58 (87)	29 (88)	29 (85)
Asian/Pacific Islander, No. (%)	3 (4)	1 (3)	2 (6)
African American, No. (%)	4 (6)	3 (9)	1 (3)
Caucasian, No. (%)	1 (1)	0 (0)	1 (3)
Other, No. (%)	1 (1)	0 (0)	1 (3)
History of skin infections	…	…	…
Yes, No. (%)	4 (6)	0 (0)	4 (12)
No, No. (%)	50 (75)	26 (79)	24 (71)
Unknown, No. (%)	13 (19)	7 (21)	6 (18)
Concomitant medications at baseline,^a^ No. (%)	23 (34)	11 (33)	12 (35)

^a^Concomitant medications in the chlorhexidine group: acetaminophen/hydrocodone (n = 1), albuterol (n = 1), dextroamphetamine (n = 1), fexofenadine (n = 1), fluocinonide (=1), fluoxetine (n = 1), folate (n = 1), glipizide (n = 1), guaifenesin (n = 1), ibuprofen (n = 7), insulin (n = 1), lamotrigine (n = 1), lisinopril (n = 1), loratadine (n = 1), and methylphenidate (n = 1); in the placebo group: albuterol (n = 2), dextroamphetamine (n = 2), dextromethorphan (n = 1), folate (n = 1), ibuprofen (n = 3), iron sulfate (n = 1), methylphenidate (n = 1), quetiapine (n = 1), sertraline (n = 1), and valproic acid (n = 1).

### *Medication* Adherence

Medication diaries were completed by 28/33 (85%) participants in the CHG group and 28/34 (82%) participants in the placebo group. Among participants with evaluable adherence data, median adherence was 100% in both groups, and mean adherence (range, SD) was 95% in the CHG group (64%–100%, 9%) and 94% in the placebo group (50%–100%, 11%).

### Oropharyngeal Decolonization

In the intention-to-treat analysis, oropharyngeal colonization at the end of treatment (EOT) visit was 45% (15/33) in the CHG group and 79% (27/34) in the placebo group (*P* = .004; difference, 34%; 95% CI, 12.2%–55.7%) (Figure 1). In the as-treated analysis, colonization was 40% (11/29) in the CHG group and 77% (23/30) in the placebo group (*P* = .003; difference, 39%; 95% CI, 15.5%–62.0%). At the 29 day follow-up visit in the ITT model, 61% (20/33) of the CHG group was positive for *S. aureus* colonization, and 85% (29/34) of the placebo group was positive for *S. aureus* colonization (*P* = .03; difference, 25%; 95% CI, 0.4%–45.2%). In the as-treated model, 54% (15/28) of the CHG group was positive for *S. aureus* colonization, and 82% (23/28) of the placebo group was positive for *S. aureus* colonization (*P* = .04; difference, 29%; 95% CI, 0.5%–51.9%).

Of the 18 CHG group participants who were oropharyngeally decolonized at EOT, 11 remained decolonized at the 29-day follow-up. In the placebo group, 2/7 (28%) decolonized participants at EOT remained decolonized at day 29. Among the 11 CHG group participants positive for *S. aureus* at EOT, 8 (72%) remained colonized at day 29. Similarly, of the 23 colonized placebo group participants at EOT, 18 (78%) remained colonized at day 29. Among CHG group participants who were oropharyngeally decolonized but nasally colonized at EOT (n = 5), 3 (60%) were oropharyngeally recolonized at day 29. Among placebo participants with the same colonization patterns at EOT (n = 6), 4 (67%) were oropharyngeally recolonized at day 29.

### Nares Colonization

At baseline, nares colonization was 39% (13/33) in the CHG group and 53% (18/34) in the placebo group (*P* = .26; difference, 14%; 95% CI, −10.1% to 37.2%). At EOT, nares colonization was 34% (10/29) in the CHG group and 53% (16/30) in the placebo group (*P* = .15; difference, 19%; 95% CI, −0.1% to 43.7%). At 29-day follow-up, nares colonization was 32% (9/28) in the CHG group and 36% (10/28) in the placebo group (*P* = .93; difference, 4%; 95% CI, −21.2% to 28.4%).

Nasal colonization dynamics are summarized alongside oropharyngeal colonization in Supplementary Figure 1 and are presented alone in Supplementary Figures 2 and 3. Among the 13 CHG-randomized children who had pretreatment nasal colonization, at EOT, among those who cleared oropharyngeal *S. aureus,* 5/18 (28%) were nasally colonized, and among those who failed to clear oropharyngeal colonization, 5/11 (45%) were nasally colonized. Among the 18 placebo-randomized children who had pretreatment nasal colonization, at EOT, among those who cleared oropharyngeal *S. aureus,* 6/7 (86%) were nasally colonized, and among those who remained oropharyngeally colonized, 10/23 (43%) were nasally colonized.

At 1-month follow-up, among those children randomized to CHG who remained oropharyngeally decolonized at both the day 8 and day 29 time points, none (0/11, 0%) were nasally colonized at day 29. Whereas among those children randomized to CHG who initially were decolonized at day 8 but were recolonized at day 29, 5/7 (71%) were nasally colonized at day 29. All children who were oropharyngeally decolonized at day 29, regardless of treatment allocation, were not nasally colonized (0/18, 0%), and among children who were oropharyngeally colonized at day 29, regardless of treatment allocation, 50% (19/38) were also nasally colonized (Supplementary Figure 1).

### Molecular Typing

Among the participants who were oropharyngeally decolonized at EOT but tested positive for *S. aureus* at the 29-day follow-up, 8/12 (66%; 4 from each of the placebo and CHG groups) exhibited a different spa type upon return. Half of these participants were recolonized oropharyngeally by a strain previously identified in their nares. The repeat patterns showed multiple differences between baseline and day 29, consistent with recolonization by a different strain rather than microvariation of the original. Among the analyzable participants with persistent oropharyngeal colonization through day 29, 16/26 (62%) retained the same strain at baseline, EOT, and day 29. Specifically, 11/17 (65%) in the placebo group and 5/8 (63%) in the CHG group maintained the same strain at all time points. Detailed spa typing data are available in Supplementary Table 1.

Because multiple participants from the same household were enrolled, we examined strain-sharing patterns among siblings as an exploratory post hoc analysis. Spa typing was conducted on oropharyngeal and nares isolates from 6 families, comprising 17 participants and with group sizes ranging from 2 to 5 children (Table 2). Siblings were not randomized to the same treatment group. In 4/6 families, siblings shared between 1 and 4 strains of *S. aureus.* At baseline, 5 pairs of siblings across 3 families had identical strains in the oropharynx and/or nares, and 3/5 pairs continued to share the same strain at day 29. Throughout the study, 3 participants acquired a strain in the oropharynx or nares that was previously identified in 1 of their siblings.

**Table 2.** *Staphylococcus aureus* Colonization Dynamics Among Siblings

Sibling/Family Group	No. of Participants in Group	Shared Strain at Baseline	Shared Strain at Day 8	Shared Strain at Day 29
A	5	Yes	No	Yes
B	2	No	No	No
C	4	No	Yes	Yes
D	2	Yes	Yes	Yes
E	2	No	No	No
F	2	Yes	No	Yes

“Shared strain” indicates that ≥1 sibling pair within the group had identical spa types detected in the oropharynx and/or nares at the specified time point. Sibling visits almost all occurred on the same date, ie, all participants from a given family had their day 1, 8, and 29 visits on the same date. Specific spa and clonal complex types are outlined in detail in Supplementary Table 1.

### Adverse Events

Overall rates of adverse events were 4/67 (6%) between the 2 groups. The most common adverse event was irritation of the mouth (2/67; 3%) between both groups. All adverse events were mild and self-limited (Table 3).

**Table 3.** Comparing Adverse Events at End of Therapy

…	0.12% CHG (n = 33), No. (%)	Placebo (n = 34), No. (%)
Staining of teeth or mouth	0 (0)	0 (0)
Increase in tooth plaque	0 (0)	0 (0)
A change in the sensation of taste	0 (0)	0 (0)
Irritation of mouth	1^c,d^ (3)	1^c,e^(3)
Aphthous ulcers	0 (0)	0 (0)
Increased redness of mouth	0 (0)	0 (0)
Peeling of the mouth of tongue	0 (0)	0 (0)
Coated tongue	0 (0)	0 (0)
Other	1^a,c,f^ (3)	1^b,c,f^ (3)

Abbreviation: CHG, chlorhexidine gluconate.

^a^participant vomited.

^b^Patient experienced a sore tongue.

^c^Degree of adverse event was mild.

^d^Adverse event resolved.

^e^Adverse event did not resolve during the study period .

## DISCUSSION

Oropharyngeal *S. aureus* colonization is increasingly recognized as a common reservoir for SSTIs in the human body, with a colonization prevalence that is similar to or exceeding that of the nares [19, 20]. However, there are very limited data on the efficacy of regimens targeted at eradication. In this double-blind, prospective randomized controlled trial, we found that a 0.12% chlorhexidine gluconate mouthwash was significantly more effective at decolonizing *S. aureus* in the oropharynx of children after 7 days of treatment compared with placebo. This difference persisted at day 29, indicating that CHG mouthwash may be a promising preventive measure against the high recurrence of SSTIs in pediatric populations.

To date, there have been only 2 other studies examining oropharyngeal *S. aureus* decolonization using CHG oral rinse or gargles. One investigation was conducted by Huang et al., who performed it as part of a post–hospital discharge MRSA decolonization strategy [24]. In this randomized clinical trial, CHG body washes, nasal mupirocin, and CHG oral gargles reduced MRSA infection compared with no decolonization. The reason why this intervention was successful and some MRSA decolonization regimens have not been [6] is probably complex and difficult to dissect, but possibly the addition of the oropharyngeal decolonization to the skin and nares was an important contributor.

In another randomized trial, Gompelman et al. evaluated topical/skin CHG decolonization plus nasal mupirocin for *S. aureus* decolonization in adults on home parenteral nutrition [27]. Only the subset of participants who were orally colonized received oropharyngeal gargles in 2 *S. aureus* decolonization regimens for adults on home parenteral nutrition [27]. A chronic regimen of CHG body washes, nasal mupirocin, +/- CHG gargles outperformed a short-term systemic antibiotic approach at whole-body *S. aureus* eradication. Given the study design, it is hard to infer the value of oral CHG gargles as only a subset of participants received it and analyses based on oral colonization were not presented.

In our study, the differences in oropharyngeal colonization between the CHG oral group and placebo gargle at EOT were significant (45% vs 79%), and they raise several interesting observations. While the CHG group decolonization rate was 55%, why 45% of children were not decolonized is not clear. First, clearly any decolonization for *S. aureus* on mucosal surfaces is far from perfect. For example, decolonization rates of nasal mupirocin are around 90% [28, 29] and have been reported to be 61% with nasal CHG [30] and 79% [31] with nasal iodophor. Besides the imperfect nature of topical decolonization of mucosal surfaces, incomplete treatment adherence could have played a role, especially given the reliance on self-reported data to measure adherence. Third, CHG resistance, likely via increased efflux pump gene carriage, has been described in *S. aureus* and may have contributed to incomplete decolonization. However, resistance has mainly been observed in health care settings with extensive use of CHG [32]. While the reasons behind decolonization failure are likely multifactorial, 15/34 (44%) and 19/38 (50%) of oropharyngeally colonized participants at day 8 and day 29, respectively, showed simultaneous nasal colonization. Moreover, 4 participants were recolonized with a strain that had previously been identified in their nares. As other studies examining *S. aureus* carriage have reported a strong association between colonization of the nares and throat [33, 34], concomitant nares decolonization may improve the persistence of oropharyngeal *S. aureus* eradication.

Consistent with the understanding that households are key reservoirs of *S. aureus* [35], only 1 sibling was successfully oropharyngeally decolonized at both day 8 and day 29, and the majority (4/6) of sibling families shared at least 1 *S. aureus* strain. While mothers are early sources of *S. aureus* transmission, the external environment plays a larger role after infancy [36]. Intrahousehold transmission may result from direct contact or fomites known to harbor *S. aureus* for extended periods [37]. Whether siblings have a genetic susceptibility to certain strains is unclear, with 1 large-scale twin study finding only a modest, insignificant influence of host genetics on *S. aureus* carriage [38]. Nevertheless, our findings support the investigation of comprehensive household decolonization strategies that target both the nares and oropharynx.

Interestingly, some participants in the placebo group were decolonized after placebo gargle (7/34 [21%] at day 8). In both the CHG and placebo groups, a subset of participants who were oropharyngeally colonized at EOT became decolonized at day 29, suggesting that some clearance occurred over time independent of the antimicrobial mechanism of CHG. There are no data to suggest one way or the other that the mechanical motion of gargling can reduce *S. aureus* carriage, and the bacterium is highly tolerant to salt within the placebo saline mouthwash [39]. As healthy individuals can exhibit intermittent *S. aureus* carriage patterns [33], the observed “decolonization” may be due to spontaneous clearance of *S. aureus* colonization over time.

This study had several limitations. One limitation is that the study population was somewhat homogeneous, as the majority of participants were Hispanic. Nevertheless, we had a wide range of ages and genders. Second, although our trial was a single-center study, it provides proof of concept as a pilot study and suggests that further research on the utility of decolonization is warranted. Third, we only did oropharyngeal decolonization and not synchronous oropharyngeal and nares decolonization, so we cannot quantify the effect of synchronous nasopharyngeal decolonization, which may be of more clinical importance. Nevertheless, our trial's findings provide important insights on the efficacy of an oropharyngeal-only decolonization regimen and the possible role nasopharyngeal colonization has on success or failure of the regimens. Fourth, our *spa* typing methods did not enable in-depth analysis of possible coexisting strains compared with whole-genome sequencing.

One strength of this study is that we performed a decolonization study in the context of a double-blind clinical trial and used an intention-to-treat model to prevent overestimation of potential treatment benefit. Second, we examined a pediatric population. Although recurrent SSTIs appear to disproportionately affect children, other oropharyngeal *S. aureus* decolonization studies have focused exclusively on adults [1, 6, 40]. Finally, we made efforts to avoid social and biologic influences to enhance clinical trial integrity, including providing new toothbrushes to minimize nonadherence.

In summary, this study found that a 7-day course of 0.12% CHG mouthwash was significantly more effective at decolonizing *S. aureus* from the oropharynx of children as compared with placebo. Future research directions should combine an oropharyngeal decolonization regimen with nasal and skin decolonization regimens to fully assess potential to decolonize patients and prevent recurrent *S. aureus* SSTI infection.

## Supplementary Material

ofag072_Supplementary_Data

## Supplementary Data

Supplementary materials are available at *Open Forum Infectious Diseases* online. Consisting of data provided by the authors to benefit the reader, the posted materials are not copyedited and are the sole responsibility of the authors, so questions or comments should be addressed to the corresponding author.
