# Supplementary material for: A Double-blind Randomized Trial of Oral Chlorhexidine Gluconate for Treatment of Oral Staphylococcus aureus Colonization in Healthy Children
Source: Open Forum Infect Dis. 2026 Feb 18;13(3):ofag072. doi: 10.1093/ofid/ofag072 (PMC12989745; doi:10.1093/ofid/ofag072)
Supplement: ofag072_Supplementary_Data [file ofag072_supplementary_data.zip › Supplementary Table 1.pdf]

Participant ID	Day	Source	Treatment	Organism	Spa Type	Clonal Complex (CC)	Spa Repeat Pattern	Notes	Legend: SA = *Staphylococcus aureus*
26	Day 1	Throat	Placebo	MSSA	2	CC 5	T1-J1-M1-B1-M1-D1-M1-G1-M1-K1		
26	Day 1	Nares		MSSA	2	CC 5	T1-J1-M1-B1-M1-D1-M1-G1-M1-K1		
26	Day 8	Throat		No SA Isolated					
26	Day 8	Nares		No SA Isolated					
26	Day 29	Throat		MSSA	151	CC 15	I2-G1-B1-B1-G1-G1-J1-A1-G1-J1		
26	Day 29	Nares		No SA Isolated					
27	Day 1	Throat	Placebo	MSSA	2	CC 5	T1-J1-M1-B1-M1-D1-M1-G1-M1-K1		
27	Day 1	Nares		MSSA	2	CC 5	T1-J1-M1-B1-M1-D1-M1-G1-M1-K1		
27	Day 8	Throat		MSSA	2	CC 5	T1-J1-M1-B1-M1-D1-M1-G1-M1-K1		
27	Day 8	Nares		MSSA	2	CC 5	T1-J1-M1-B1-M1-D1-M1-G1-M1-K1		
27	Day 29	Throat		MSSA	2	CC 5	T1-J1-M1-B1-M1-D1-M1-G1-M1-K1		
27	Day 29	Nares		No SA Isolated					
29	Day 1	Throat	Chlorhexidine	MSSA	33	CC 30	W1-G1-K1-A1-K1-A1-O1-M1-Q1-Q1	Sibling (Family K)	
29	Day 1	Nares		MSSA	1619	Unknown	K6-D1-L1-L6-M1-M1		
29	Day 8	Throat		MSSA	16	CC 30	W1-G1-K1-A1-K1-A1-O1-M1-Q1-Q1-Q1		
29	Day 8	Nares		MSSA	33	CC 30	W1-G1-K1-A1-K1-A1-O1-M1-Q1-Q1		
29	Day 29	Throat		MSSA	33	CC 30	W1-G1-K1-A1-K1-A1-O1-M1-Q1-Q1		
29	Day 29	Nares		No SA Isolated					
32	Day 1	Throat	Chlorhexidine	MSSA	193	CC 72	U1-J1-G1-F1-G1-M1-D1-M1-G1-G1-M1		
32	Day 1	Nares		No SA Isolated					
32	Day 8	Throat		MSSA	193	CC 72	U1-J1-G1-F1-G1-M1-D1-M1-G1-G1-M1		
32	Day 8	Nares		No SA Isolated					
32	Day 29	Throat		MSSA	193	CC 72	U1-J1-G1-F1-G1-M1-D1-M1-G1-G1-M1		
32	Day 29	Nares		No SA Isolated					
33	Day 1	Throat	Placebo	MSSA	92	CC 8	Y1-C2-F1-M1-B1-Q1-B1-L1-O1	Isolate missing	
33	Day 1	Nares		MSSA					
33	Day 8	Throat		MSSA	154	CC 8	Y1-C2-F1-M1-B1-Q1-B1-L1-O1-O1		
33	Day 8	Nares		MSSA	New	CC 182 Probable	Z1-B1-L1-M1-J1-Q1-Q1		
33	Day 29	Throat		MSSA	154	CC 8	Y1-C2-F1-M1-B1-Q1-B1-L1-O1-O1		
33	Day 29	Nares		MSSA	New	CC 182 Probable	Z1-B1-L1-M1-J1-Q1-Q1		

34	Day 1	Throat	Chlorhexidine	MSSA				Isolate missing
34	Day 1	Nares		No SA Isolated				Sibling (Family X)
34	Day 8	Throat		No SA Isolated				Sibling (Family X)
34	Day 29	Throat		No SA Isolated				Sibling (Family X)
34	Day 29	Nares		No SA Isolated				Sibling (Family X)
35	Day 1	Throat	Placebo	MSSA	151	CC 15	I2-G1-B1-B1-G1-G1-J1-A1-G1-J1	Sibling (Family X)
35	Day 1	Nares		MSSA	1619	unknown	K6-D1-L1-L6-M1-M1	Sibling (Family X)
35	Day 8	Throat						Participant lost to follow up
35	Day 8	Nares						Participant lost to follow up
35	Day 29	Throat						Participant lost to follow up
35	Day 29	Nares						Participant lost to follow up
36	Day 1	Throat	Chlorhexidine	MSSA	1619	unknown	K6-D1-L1-L6-M1-M1	Sibling (Family X)
36	Day 1	Nares		No SA Isolated				
36	Day 8	Throat		No SA Isolated				
36	Day 8	Nares		No SA Isolated				
36	Day 29	Throat		No SA Isolated				
36	Day 29	Nares		No SA Isolated				
37	Day 1	Throat	Placebo	MSSA	151	CC 15	I2-G1-B1-B1-G1-G1-J1-A1-G1-J1	Sibling (Family A)
37	Day 1	Nares		No SA Isolated				
37	Day 8	Throat		MSSA	122	CC 188	U1-J1-G1-F1-M1-B1	
37	Day 8	Nares		No SA Isolated				
37	Day 29	Throat		No SA Isolated				
37	Day 29	Nares		No SA Isolated				
38	Day 1	Throat	Chlorhexidine	MSSA	2	CC 5	T1-J1-M1-B1-M1-D1-M1-G1-M1-K1	Sibling (Family A)
38	Day 1	Nares		No SA Isolated				
38	Day 8	Throat		No SA Isolated				
38	Day 8	Nares		No SA Isolated				
38	Day 29	Throat		No SA Isolated				
38	Day 29	Nares		No SA Isolated				
39	Day 1	Throat	Chlorhexidine	MSSA	1	CC 8	Y1-H1-G1-F1-M1-B1-Q1-B1-L1-O1	Sibling (Family A)
39	Day 1	Nares		No SA Isolated				
39	Day 8	Throat		MSSA	1	CC 8	Y1-H1-G1-F1-M1-B1-Q1-B1-L1-O1	
39	Day 8	Nares		No SA Isolated				
39	Day 29	Throat		No SA Isolated				
39	Day 29	Nares		No SA Isolated				

40	Day 1	Throat	Chlorhexidine	MSSA	1	CC 8	Y1-H1-G1-F1-M1-B1-Q1-B1-L1-O1	Sibling (Family A)
40	Day 1	Nares		MSSA	New	Unknown	Z1-B1-G1	
40	Day 8	Throat		No SA Isolated				
40	Day 8	Nares		No SA Isolated				
40	Day 29	Throat		MSSA	New	Unknown	Z1-B1-G1	
40	Day 29	Nares		MSSA	New	Unknown	Z1-B1-G1	
41	Day 1	Throat	Chlorhexidine	MSSA	151	CC 15	I2-G1-B1-B1-G1-G1-J1-A1-G1-J1	Sibling (Family A)
41	Day 1	Nares		MSSA	New	Unknown	Z1-B1-G1	
41	Day 8	Throat		No SA Isolated				
41	Day 8	Nares		No SA Isolated				
41	Day 29	Throat		MSSA	2	CC 5	T1-J1-M1-B1-M1-D1-M1-G1-M1-K1	
41	Day 29	Nares		MSSA	New	Unknown	Z1-B1-G1	
42	Day 1	Throat	Placebo	MSSA	2	CC 5	T1-J1-M1-B1-M1-D1-M1-G1-M1-K1	
42	Day 1	Nares		MSSA	1619	Unknown	K6-D1-L1-L6-M1-M1	
42	Day 8	Throat		MSSA	17	CC 59	Z1-D1-M1-D1-M1-N1-K1-B1	
42	Day 8	Nares		No SA Isolated				
42	Day 29	Throat		No SA Isolated				
42	Day 29	Nares		No SA Isolated				
43	Day 1	Throat	Placebo	MSSA	768	CC 12	U1-J1-F1-P1-L1-M1	
43	Day 1	Nares		MSSA	1619	Unknown	K6-D1-L1-L6-M1-M1	
43	Day 8	Throat						Participant lost to follow up
43	Day 8	Nares						Participant lost to follow up
43	Day 29	Throat						Participant lost to follow up
43	Day 29	Nares						Participant lost to follow up
47	Day 1	Throat	Placebo	MSSA	New	Unknown	Y1-H1-O1-O1	
47	Day 1	Nares		No SA Isolated				
47	Day 8	Throat						Participant lost to follow up
47	Day 8	Nares						Participant lost to follow up
47	Day 29	Throat						Participant lost to follow up
47	Day 29	Nares						Participant lost to follow up
48	Day 1	Throat	Placebo	MSSA	11	CC 5	T1-J1-M1-B1-M1-D1-M1-M1-K1	
48	Day 1	Nares		No SA Isolated				
48	Day 8	Throat		MSSA	11	CC 5	T1-J1-M1-B1-M1-D1-M1-M1-K1	
48	Day 8	Nares		No SA Isolated				
48	Day 29	Throat		MSSA	11	CC 5	T1-J1-M1-B1-M1-D1-M1-M1-K1	
48	Day 29	Nares		MSSA	11	CC 5	T1-J1-M1-B1-M1-D1-M1-M1-K1	

50	Day 1	Throat	Chlorhexidine	MSSA	122	CC 188	U1-J1-G1-F1-M1-B1	
50	Day 1	Nares		No SA Isolated				
50	Day 8	Throat		No SA Isolated				
50	Day 8	Nares		No SA Isolated				
50	Day 29	Throat		MSSA	1619	Unknown	K6-D1-L1-L6-M1-M1	
50	Day 29	Nares		No SA Isolated				
55	Day 1	Throat	Placebo	MSSA	62	CC 45	A2-A1-K1-E1-M1-B1-K1-B1	Sibling (Family B)
55	Day 1	Nares		No SA Isolated				
55	Day 8	Throat		MSSA	62	CC 45	A2-A1-K1-E1-M1-B1-K1-B1	
55	Day 8	Nares		MSSA	62	CC 45	A2-A1-K1-E1-M1-B1-K1-B1	
55	Day 29	Throat		MSSA	230	CC 5	T1-M1-B1-M1-D1-M1-G1-M1-K1	
55	Day 29	Nares		MSSA	62	CC 45	A2-A1-K1-E1-M1-B1-K1-B1	
56	Day 1	Throat	Placebo	MSSA	105	CC 97	U1-J1-G1-F1-M1-B1-B1-B1-P1-B1	Sibling (Family B)
56	Day 1	Nares		MRSA	985	CC 8	Z1-F1-M1-B1-Q1-B1-L1-O1	
56	Day 8	Throat		No SA Isolated				
56	Day 8	Nares		MSSA	New	Unknown	Z1-Q1-N1-G1-K1-B1-K1-G1-P1-B1	
56	Day 29	Throat		MRSA	985	CC 8	Z1-F1-M1-B1-Q1-B1-L1-O1	
56	Day 29	Nares		No SA Isolated				
57	Day 1	Throat	Chlorhexidine	MSSA	1	CC 8	Y1-H1-G1-F1-M1-B1-Q1-B1-L1-O1	
57	Day 1	Nares		No SA Isolated				
57	Day 8	Throat						Participant lost to follow up
57	Day 8	Nares						Participant lost to follow up
57	Day 29	Throat						Participant lost to follow up
57	Day 29	Nares						Participant lost to follow up
58	Day 1	Throat	Chlorhexidine	MSSA	New	CC 5	T1-J1-M1-B1-M1-M1-D1-M1-G1-M1-K1	Sibling (Family C)
58	Day 1	Nares		No SA Isolated				
58	Day 8	Throat		No SA Isolated				
58	Day 8	Nares		MSSA	541	CC 45 Probable	X1-K1-B1-B3	
58	Day 29	Throat		MSSA	New	CC 5	T1-J1-M1-B1-M1-M1-D1-M1-G1-M1-K1	
58	Day 29	Nares		MSSA	541	CC 45 Probable	X1-K1-B1-B3	
61	Day 1	Throat	Placebo	MSSA	New	Unknown	T1-J1-M1-B1-M1	Sibling (Family C)
61	Day 1	Nares		MSSA	1619	Unknown	K6-D1-L1-L6-M1-M1	
61	Day 8	Throat		MSSA	New	Unknown	T1-J1-M1-B1-M1	
61	Day 8	Nares		MSSA	New	Unknown	T1-J1-M1-B1-M1	
61	Day 29	Throat		MSSA	New	Unknown	T1-J1-M1-B1-M1	
61	Day 29	Nares		MSSA	New	Unknown	T1-J1-M1-B1-M1	
62	Day 1	Throat	Placebo	MSSA	33	CC 30	W1-G1-K1-A1-K1-A1-O1-M1-Q1-Q1	Sibling (Family C)
62	Day 1	Nares		MSSA	541	CC 45 Probable	X1-K1-B1-B3	
62	Day 8	Throat		No SA Isolated				
62	Day 8	Nares		MSSA	541	CC 45 Probable	X1-K1-B1-B3	
62	Day 29	Throat		MSSA	541	CC 45 Probable	X1-K1-B1-B3	
62	Day 29	Nares		MSSA	541	CC 45 Probable	X1-K1-B1-B3	

63	Day 1	Throat	Placebo	MSSA	42	CC 45	A2-A1-K1-B1-E1-M1-B1-K1-B1	Sibling (Family C)
63	Day 1	Nares		MSSA	New	CC 72	U1-J1-G1-F1-G1-M1-D1-M1-G1-G1-G1-M1-M1	
63	Day 8	Throat		MSSA	New	CC 72	U1-J1-G1-F1-G1-M1-D1-M1-G1-G1-G1-M1-M1	
63	Day 8	Nares		MSSA	1619	Unknown	K6-D1-L1-L6-M1-M1	
63	Day 29	Throat		MSSA	New	CC 72	U1-J1-G1-F1-G1-M1-D1-M1-G1-G1-G1-M1-M1	
63	Day 29	Nares		MSSA	New	CC 72	U1-J1-G1-F1-G1-M1-D1-M1-G1-G1-G1-M1-M1	
66	Day 1	Throat	Placebo	MSSA	New	CC 5	T1-J1-M1-B1-M1-D1-M1-G1-G1	
66	Day 1	Nares		No SA Isolated				
66	Day 8	Throat		No SA Isolated				
66	Day 8	Nares		MRSA	985	CC 8	Z1-F1-M1-B1-Q1-B1-L1-O1	
66	Day 29	Throat		MRSA	985	CC 8	Z1-F1-M1-B1-Q1-B1-L1-O1	
66	Day 29	Nares		MRSA	985	CC 8	Z1-F1-M1-B1-Q1-B1-L1-O1	
67	Day 1	Throat	Chlorhexidine	MSSA	New	CC 45	A2-A1-K1-E1-E1-E1-M1-E1-K1-B1	
67	Day 1	Nares		No SA Isolated				
67	Day 8	Throat		No SA Isolated				
67	Day 8	Nares		No SA Isolated				
67	Day 29	Throat		No SA Isolated				
67	Day 29	Nares		No SA Isolated				
68	Day 1	Throat	Chlorhexidine	MSSA	17	CC 59	Z1-D1-M1-D1-M1-N1-K1-B1	
68	Day 1	Nares		MSSA	17	CC 59	Z1-D1-M1-D1-M1-N1-K1-B1	
68	Day 8	Throat						Participant lost to follow up
68	Day 8	Nares						Participant lost to follow up
68	Day 29	Throat						Participant lost to follow up
68	Day 29	Nares						Participant lost to follow up
71	Day 1	Throat	Chlorhexidine	MSSA	New	Unknown	V5-N1-O1-M1-M1-M1-K1-K1-K1	
71	Day 1	Nares		No SA Isolated				
71	Day 8	Throat						Participant lost to follow up
71	Day 8	Nares						Participant lost to follow up
71	Day 29	Throat						Participant lost to follow up
71	Day 29	Nares						Participant lost to follow up
73	Day 1	Throat	Placebo	MRSA	17	CC 59	Z1-D1-M1-D1-M1-N1-K1-B1	
73	Day 1	Nares		MSSA	1619	Unknown	K6-D1-L1-L6-M1-M1	
73	Day 8	Throat		No SA Isolated				
73	Day 8	Nares		MSSA	1619	Unknown	K6-D1-L1-L6-M1-M1	
73	Day 29	Throat		MRSA	17	CC 59	Z1-D1-M1-D1-M1-N1-K1-B1	
73	Day 29	Nares		No SA Isolated				
77	Day 1	Throat	Chlorhexidine	MRSA	2	CC 5	T1-J1-M1-B1-M1-D1-M1-G1-M1-K1	
77	Day 1	Nares		No SA Isolated				
77	Day 8	Throat		MRSA	2	CC 5	T1-J1-M1-B1-M1-D1-M1-G1-M1-K1	
77	Day 8	Nares		No SA Isolated				
77	Day 29	Throat		No SA Isolated				
77	Day 29	Nares		No SA Isolated				

79	Day 1	Throat	Placebo	MSSA	122	CC 188	U1-J1-G1-F1-M1-B1	Sibling (Family E)
79	Day 1	Nares		No SA Isolated				
79	Day 8	Throat		MSSA	106	CC 188	U1-J1-G1-F1-M1-B1-B1	
79	Day 8	Nares		No SA Isolated				
79	Day 29	Throat		MSSA	122	CC 188	U1-J1-G1-F1-M1-B1	
79	Day 29	Nares		No SA Isolated				
80	Day 1	Throat	Chlorhexidine	MSSA	23	CC 5	T1-J1-M1-B1-M1-D1-M1-G1-K1	
80	Day 1	Nares		No SA Isolated				
80	Day 8	Throat		No SA Isolated				
80	Day 8	Nares		No SA Isolated				
80	Day 29	Throat		No SA Isolated				
80	Day 29	Nares		No SA Isolated				
82	Day 1	Throat	Placebo	MSSA	2	CC 5	T1-J1-M1-B1-M1-D1-M1-G1-M1-K1	Sibling (Family D)
82	Day 1	Nares		MSSA	2	CC 5	T1-J1-M1-B1-M1-D1-M1-G1-M1-K1	
82	Day 8	Throat		MSSA	2	CC 5	T1-J1-M1-B1-M1-D1-M1-G1-M1-K1	
82	Day 8	Nares		MSSA	2	CC 5	T1-J1-M1-B1-M1-D1-M1-G1-M1-K1	
82	Day 29	Throat		MSSA	2	CC 5	T1-J1-M1-B1-M1-D1-M1-G1-M1-K1	
82	Day 29	Nares		MSSA	2	CC 5	T1-J1-M1-B1-M1-D1-M1-G1-M1-K1	
83	Day 1	Throat	Chlorhexidine	MSSA	2	CC 5	T1-J1-M1-B1-M1-D1-M1-G1-M1-K1	Sibling (Family D)
83	Day 1	Nares		MSSA	2	CC 5	T1-J1-M1-B1-M1-D1-M1-G1-M1-K1	
83	Day 8	Throat		No SA Isolated				
83	Day 8	Nares		MSSA	2	CC 5	T1-J1-M1-B1-M1-D1-M1-G1-M1-K1	
83	Day 29	Throat		MSSA	122	CC 188	U1-J1-G1-F1-M1-B1	
83	Day 29	Nares		MSSA	2	CC 5	T1-J1-M1-B1-M1-D1-M1-G1-M1-K1	
86	Day 1	Throat	Chlorhexidine	MSSA	17	CC 59	Z1-D1-M1-D1-M1-N1-K1-B1	Sibling (Family E)
86	Day 1	Nares		MSSA	17	CC 59	Z1-D1-M1-D1-M1-N1-K1-B1	
86	Day 8	Throat		MSSA	17	CC 59	Z1-D1-M1-D1-M1-N1-K1-B1	
86	Day 8	Nares		MSSA	17	CC 59	Z1-D1-M1-D1-M1-N1-K1-B1	
86	Day 29	Throat		MSSA	17	CC 59	Z1-D1-M1-D1-M1-N1-K1-B1	
86	Day 29	Nares		MSSA	17	CC 59	Z1-D1-M1-D1-M1-N1-K1-B1	
88	Day 1	Throat	Placebo	MSSA	47	CC 5	T1-M1-D1-M1-G1-M1-K1	
88	Day 1	Nares		MSSA	47	CC 5	T1-M1-D1-M1-G1-M1-K1	
88	Day 8	Throat		MSSA	47	CC 5	T1-M1-D1-M1-G1-M1-K1	
88	Day 8	Nares		MSSA	47	CC 5	T1-M1-D1-M1-G1-M1-K1	
88	Day 29	Throat		No SA Isolated				
88	Day 29	Nares		No SA Isolated				
89	Day 1	Throat	Placebo	MSSA	1376	CC 398	X1-K1-A1-O1-B1-O1	
89	Day 1	Nares		MSSA	1376	CC 398	X1-K1-A1-O1-B1-O1	
89	Day 8	Throat		MSSA	65	CC 5	T1-J1-M1-B1-M1-D1-M1-G1-M1-M1-K1	
89	Day 8	Nares		MSSA	1376	CC 398	X1-K1-A1-O1-B1-O1	
89	Day 29	Throat		MSSA	1376	CC 398	X1-K1-A1-O1-B1-O1	
89	Day 29	Nares		MSSA	1376	CC 398	X1-K1-A1-O1-B1-O1	

94	Day 1	Throat	Placebo	MSSA	1629	CC 25	Z1-F1-G1-M1-D1-M1-G1-M1	
94	Day 1	Nares		No SA Isolated				
94	Day 8	Throat		MSSA	946	CC 25	Z1-F1-G1-M1-D1-M1-G1-G1-M1-G1-G1-M1	
94	Day 8	Nares		No SA Isolated				
94	Day 29	Throat		MSSA	565	Unknown	Z1-P1-N1-G1-K1-B1-K1-G1-P1-B1	
94	Day 29	Nares		No SA Isolated				
98	Day 1	Throat	Chlorhexidine	MSSA	1	CC 8	Y1-H1-G1-F1-M1-B1-Q1-B1-L1-O1	
98	Day 1	Nares		MSSA	1619	Unknown	K6-D1-L1-L6-M1-M1	
98	Day 8	Throat		No SA Isolated				
98	Day 8	Nares		MSSA	1	CC 8	Y1-H1-G1-F1-M1-B1-Q1-B1-L1-O1	
98	Day 29	Throat		MSSA	1	CC 8	Y1-H1-G1-F1-M1-B1-Q1-B1-L1-O1	
98	Day 29	Nares		MSSA	1	CC 8	Y1-H1-G1-F1-M1-B1-Q1-B1-L1-O1	
99	Day 1	Throat	Chlorhexidine	MSSA	175	CC 1	U1-J1-F1-K1-P1-E1	
99	Day 1	Nares		No SA Isolated				
99	Day 8	Throat		No SA Isolated				
99	Day 8	Nares		No SA Isolated				
99	Day 29	Throat		No SA Isolated				
99	Day 29	Nares		No SA Isolated				
103	Day 1	Throat	Chlorhexidine	MSSA	33	CC 30	W1-G1-K1-A1-K1-A1-O1-M1-Q1-Q1	
103	Day 1	Nares		MSSA	33	CC 30	W1-G1-K1-A1-K1-A1-O1-M1-Q1-Q1	
103	Day 8	Throat		No SA Isolated				
103	Day 8	Nares		MSSA	33	CC 30	W1-G1-K1-A1-K1-A1-O1-M1-Q1-Q1	
103	Day 29	Throat		No SA Isolated				
103	Day 29	Nares		No SA Isolated				
106	Day 1	Throat	Placebo	MSSA	91	CC 8	Y1-C2-F1-M1-B1-Q1-B1-L1-O1	
106	Day 1	Nares		No SA Isolated				
106	Day 8	Throat		MSSA				Isolate missing
106	Day 8	Nares		No SA Isolated				
106	Day 29	Throat		MSSA	91	CC 8	Y1-C2-F1-M1-B1-Q1-B1-L1-O1	
106	Day 29	Nares		No SA Isolated				
107	Day 1	Throat	Placebo	MSSA	193	CC 72	U1-J1-G1-F1-G1-M1-D1-M1-G1-G1-M1	
107	Day 1	Nares		MSSA	193	CC 72	U1-J1-G1-F1-G1-M1-D1-M1-G1-G1-M1	
107	Day 8	Throat		MSSA	193	CC 72	U1-J1-G1-F1-G1-M1-D1-M1-G1-G1-M1	
107	Day 29	Nares		No SA Isolated				
107	Day 29	Throat		MSSA	193	CC 72	U1-J1-G1-F1-G1-M1-D1-M1-G1-G1-M1	
107	Day 29	Nares		No SA Isolated				
109	Day 1	Throat	Chlorhexidine	MSSA	122	CC 188	U1-J1-G1-F1-M1-B1	
109	Day 1	Nares		No SA Isolated				
109	Day 8	Throat		No SA Isolated				
109	Day 8	Nares		No SA Isolated				
109	Day 29	Throat		No SA Isolated				
109	Day 29	Nares		No SA Isolated				

110	Day 1	Throat	Chlorhexidine	MSSA	1232	CC 45	X1-K1-A1-K1-B1-B1-K1-B1	
110	Day 1	Nares	Chlorhexidine	No SA Isolated				
110	Day 8	Throat	Chlorhexidine	No SA Isolated				
110	Day 8	Nares	Chlorhexidine	No SA Isolated				
110	Day 29	Throat	Chlorhexidine	No SA Isolated				
110	Day 29	Nares	Chlorhexidine	No SA Isolated				
115	Day 1	Throat	Placebo	MSSA	New	Unknown	U1-M1-B1-B1-P1-B1	
115	Day 1	Nares	Placebo	No SA Isolated				
115	Day 8	Throat	Placebo	No SA Isolated				
115	Day 8	Nares	Placebo	MSSA	New	Unknown	U1-M1-B1-B1-P1-B1	
115	Day 29	Throat	Placebo	No SA Isolated				
115	Day 29	Nares	Placebo	No SA Isolated				
119	Day 1	Throat	Placebo	MSSA	175	CC 1	U1-J1-F1-K1-P1-E1	
119	Day 1	Nares	Placebo	MSSA	16	CC 30	W1-G1-K1-A1-K1-A1-O1-M1-Q1-Q1-Q1	
119	Day 8	Throat	Placebo	MSSA	New	CC 182 Probable	Z1-B1-L1-M1-J1-Q1-Q1	
119	Day 8	Nares	Placebo	No SA Isolated				
119	Day 29	Throat	Placebo	MSSA	New	CC 182 Probable	Z1-B1-L1-M1-J1-Q1-Q1	
119	Day 29	Nares	Placebo	No SA Isolated				
126	Day 1	Throat	Chlorhexidine	MSSA	New	CC 182 Probable	Z1-B1-L1-M1-J1-Q1-Q1	
126	Day 1	Nares	Chlorhexidine	MSSA	New	CC 182 Probable	Z1-B1-L1-M1-J1-Q1-Q1	
126	Day 8	Throat	Chlorhexidine	No SA Isolated				
126	Day 8	Nares	Chlorhexidine	MSSA	175	CC 1	U1-J1-F1-K1-P1-E1	
126	Day 29	Throat	Chlorhexidine					Isolate missing
126	Day 29	Nares	Chlorhexidine					Isolate missing
132	Day 1	Throat	Placebo	MSSA	New	CC 188 Probable	T1-J1-G1-F1-M1-B1	
132	Day 1	Nares	Placebo	MSSA	New	CC 188 Probable	T1-J1-G1-F1-M1-B1	
132	Day 8	Throat	Placebo	No SA Isolated				
132	Day 8	Nares	Placebo	MSSA	New	CC 188 Probable	T1-J1-G1-F1-M1-B1	
132	Day 29	Throat	Placebo	No SA Isolated				
132	Day 29	Nares	Placebo	No SA Isolated				
134	Day 1	Throat	Chlorhexidine	MSSA	1037	CC 8	Y1-H1-G1-F1-M1-B1-O1	
134	Day 1	Nares	Chlorhexidine	MSSA	1037	CC 8	Y1-H1-G1-F1-M1-B1-O1	
134	Day 8	Throat	Chlorhexidine	MSSA	1037	CC 8	Y1-H1-G1-F1-M1-B1-O1	
134	Day 8	Nares	Chlorhexidine	MSSA	1037	CC 8	Y1-H1-G1-F1-M1-B1-O1	
134	Day 29	Throat	Chlorhexidine	MSSA	1037	CC 8	Y1-H1-G1-F1-M1-B1-O1	
134	Day 29	Nares	Chlorhexidine	MSSA	1037	CC 8	Y1-H1-G1-F1-M1-B1-O1	
135	Day 1	Throat	Chlorhexidine	MSSA	New	CC 188 Probable	T1-J1-G1-F1-M1-B1	
135	Day 1	Nares	Chlorhexidine	No SA Isolated				
135	Day 8	Throat	Chlorhexidine	No SA Isolated				
135	Day 8	Nares	Chlorhexidine	No SA Isolated				
135	Day 29	Throat	Chlorhexidine	No SA Isolated				
135	Day 29	Nares	Chlorhexidine	No SA Isolated				

142	Day 1	Throat	Placebo	MSSA	468	CC 30	X1-K1-A1-K1-A1-O1-M1-Q1-Q1	
142	Day 1	Nares		No SA Isolated				
142	Day 8	Throat						Participant lost to follow up
142	Day 8	Nares						Participant lost to follow up
142	Day 29	Throat						Participant lost to follow up
142	Day 29	Nares						Participant lost to follow up
145	Day 1	Throat	Placebo	MSSA	2	CC 5	T1-J1-M1-B1-M1-D1-M1-G1-M1-K1	
145	Day 1	Nares		No SA Isolated				
145	Day 8	Throat		MSSA	2	CC 5	T1-J1-M1-B1-M1-D1-M1-G1-M1-K1	
145	Day 8	Nares		No SA Isolated				
145	Day 29	Throat		MSSA	2	CC 5	T1-J1-M1-B1-M1-D1-M1-G1-M1-K1	
145	Day 29	Nares		No SA Isolated				
150	Day 1	Throat	Placebo	MSSA	370	CC 5	T1-J1-M1-B1-M1-D1-M1-G1-G1-G1-K1	Sibling (Family U)
150	Day 1	Nares		No SA Isolated				Sibling (Family U)
150	Day 8	Throat		MSSA	370	CC 5	T1-J1-M1-B1-M1-D1-M1-G1-G1-G1-K1	Sibling (Family U)
150	Day 8	Nares		No SA Isolated				Sibling (Family U)
150	Day 29	Throat						Participant lost to follow up
150	Day 29	Nares						Participant lost to follow up
155	Day 1	Throat	Placebo	MSSA	370	CC 5	T1-J1-M1-B1-M1-D1-M1-G1-G1-G1-K1	Sibling (Family U)
155	Day 1	Nares		No SA Isolated				Sibling (Family U)
155	Day 8	Throat		MSSA	370	CC 5	T1-J1-M1-B1-M1-D1-M1-G1-G1-G1-K1	Sibling (Family U)
155	Day 8	Nares		MSSA	370	CC 5	T1-J1-M1-B1-M1-D1-M1-G1-G1-G1-K1	Sibling (Family U)
155	Day 29	Throat						Participant lost to follow up
155	Day 29	Nares						Participant lost to follow up
156	Day 1	Throat	Chlorhexidine	MSSA	370	CC 5	T1-J1-M1-B1-M1-D1-M1-G1-G1-G1-K1	Sibling (Family U)
156	Day 1	Nares		No SA Isolated				Sibling (Family U)
156	Day 8	Throat		MSSA	370	CC 5	T1-J1-M1-B1-M1-D1-M1-G1-G1-G1-K1	Sibling (Family U)
156	Day 8	Nares		No SA Isolated				Sibling (Family U)
156	Day 29	Throat						Participant lost to follow up
156	Day 29	Nares						Participant lost to follow up
157	Day 1	Throat	Chlorhexidine	MRSA				Data non-analyzable; participant left building before study drug administration
157	Day 1	Nares						
157	Day 8	Throat						
157	Day 8	Nares						
157	Day 29	Throat						
157	Day 29	Nares						
167	Day 1	Throat	Chlorhexidine	MSSA	176	CC 5	T1-J1-M1-B1-M1-K1	
167	Day 1	Nares		No SA Isolated				
167	Day 8	Throat						Participant lost to follow up
167	Day 8	Nares						Participant lost to follow up
167	Day 29	Throat						Participant lost to follow up
167	Day 29	Nares						Participant lost to follow up

168	Day 1	Throat	Placebo	MSSA	214	CC 30	W1-G1-K1-A1-M1-Q1-Q1	
168	Day 1	Nares		No SA Isolated				
168	Day 8	Throat		MSSA	214	CC 30	W1-G1-K1-A1-M1-Q1-Q1	
168	Day 8	Nares		No SA Isolated				
168	Day 29	Throat		MSSA	214	CC 30	W1-G1-K1-A1-M1-Q1-Q1	
168	Day 29	Nares		No SA Isolated				
172	Day 1	Throat	Chlorhexidine	MSSA	105	CC 97	U1-J1-G1-F1-M1-B1-B1-B1-P1-B1	
172	Day 1	Nares		MSSA	105	CC 97	U1-J1-G1-F1-M1-B1-B1-B1-P1-B1	
172	Day 8	Throat		MSSA	105	CC 97	U1-J1-G1-F1-M1-B1-B1-B1-P1-B1	
172	Day 8	Nares		MSSA	105	CC 97	U1-J1-G1-F1-M1-B1-B1-B1-P1-B1	
172	Day 29	Throat		MSSA	105	CC 97	U1-J1-G1-F1-M1-B1-B1-B1-P1-B1	
172	Day 29	Nares		MSSA	105	CC 97	U1-J1-G1-F1-M1-B1-B1-B1-P1-B1	
174	Day 1	Throat	Placebo	MSSA	1513	CC 72 Probable	U1-J1-G1-F1-G1-U2-D1-M1-G1-G1-M1	Sibling (Family F)
174	Day 1	Nares		MSSA	1513	CC 72 Probable	U1-J1-G1-F1-G1-U2-D1-M1-G1-G1-M1	
174	Day 8	Throat		MSSA	1513	CC 72 Probable	U1-J1-G1-F1-G1-U2-D1-M1-G1-G1-M1	
174	Day 8	Nares		MSSA	1513	CC 72 Probable	U1-J1-G1-F1-G1-U2-D1-M1-G1-G1-M1	
174	Day 29	Throat		MSSA	1513	CC 72 Probable	U1-J1-G1-F1-G1-U2-D1-M1-G1-G1-M1	
174	Day 29	Nares		MSSA	1513	CC 72 Probable	U1-J1-G1-F1-G1-U2-D1-M1-G1-G1-M1	
175	Day 1	Throat	Chlorhexidine	MSSA	1513	CC 72 Probable	U1-J1-G1-F1-G1-U2-D1-M1-G1-G1-M1	Sibling (Family F)
175	Day 1	Nares		No SA Isolated				
175	Day 8	Throat		No SA Isolated				
175	Day 8	Nares		No SA Isolated				
175	Day 29	Throat		MSSA	1513	CC 72 Probable	U1-J1-G1-F1-G1-U2-D1-M1-G1-G1-M1	
175	Day 29	Nares		No SA Isolated				
177	Day 1	Throat	Chlorhexidine	MRSA	21	CC 15	U1-J1-G1-B1-B1-G1-G1-J1-A1-G1-J1	
177	Day 1	Nares		MRSA	1	CC 8	Y1-H1-G1-F1-M1-B1-Q1-B1-L1-O1	
177	Day 8	Throat		MRSA	1	CC 8	Y1-H1-G1-F1-M1-B1-Q1-B1-L1-O1	
177	Day 8	Nares		No SA Isolated				
177	Day 29	Throat		MRSA	21	CC 15	U1-J1-G1-B1-B1-G1-G1-J1-A1-G1-J1	
177	Day 29	Nares		No SA Isolated				
185	Day 1	Throat	Chlorhexidine	MRSA	468	CC 30	X1-K1-A1-K1-A1-O1-M1-Q1-Q1	
185	Day 1	Nares		MSSA	1376	CC 398	X1-K1-A1-O1-B1-O1	
185	Day 8	Throat		MRSA	77	unknown	Y1-H1-O1	
185	Day 8	Nares		MSSA	1376	CC 398	X1-K1-A1-O1-B1-O1	
185	Day 29	Throat		MRSA	1	CC 8	Y1-H1-G1-F1-M1-B1-Q1-B1-L1-O1	
185	Day 29	Nares		MSSA	33	CC 30	W1-G1-K1-A1-K1-A1-O1-M1-Q1-Q1	
186	Day 1	Throat	Placebo	MSSA	2	CC 5	T1-J1-M1-B1-M1-D1-M1-G1-M1-K1	
186	Day 1	Nares		No SA Isolated				
186	Day 8	Throat		MSSA	2	CC 5	T1-J1-M1-B1-M1-D1-M1-G1-M1-K1	
186	Day 8	Nares		No SA Isolated				
186	Day 29	Throat		MSSA	2	CC 5	T1-J1-M1-B1-M1-D1-M1-G1-M1-K1	
186	Day 29	Nares		No SA Isolated				

187	Day 1	Throat	Placebo	MSSA	17	CC 59	Z1-D1-M1-D1-M1-N1-K1-B1	
187	Day 1	Nares		No SA Isolated				
187	Day 8	Throat		MSSA	New	CC 15 Probable	U1-J1-G1-B1-G1-W2-J1-A1-G1-J1	
187	Day 8	Nares		No SA Isolated				
187	Day 29	Throat		MSSA	New	CC 15 Probable	U1-J1-G1-B1-G1-W2-J1-A1-G1-J1	
187	Day 29	Nares		No SA Isolated				
189	Day 1	Throat	Chlorhexidine	MSSA	363	CC 8	Y1-G1-F1-M1-B1-Q1-B1-L1-O1	
189	Day 1	Nares		No SA Isolated				
189	Day 8	Throat		MSSA	363	CC 8	Y1-G1-F1-M1-B1-Q1-B1-L1-O1	
189	Day 8	Nares		No SA Isolated				
189	Day 29	Throat		MSSA	46	CC 8	Y1-M1-B1-Q1-B1-L1-O1	
189	Day 29	Nares		No SA Isolated				
