# Supplementary figures and images for: A Double-blind Randomized Trial of Oral Chlorhexidine Gluconate for Treatment of Oral Staphylococcus aureus Colonization in Healthy Children
Source: Open Forum Infect Dis. 2026 Feb 18;13(3):ofag072. doi: 10.1093/ofid/ofag072 (PMC12989745; doi:10.1093/ofid/ofag072)

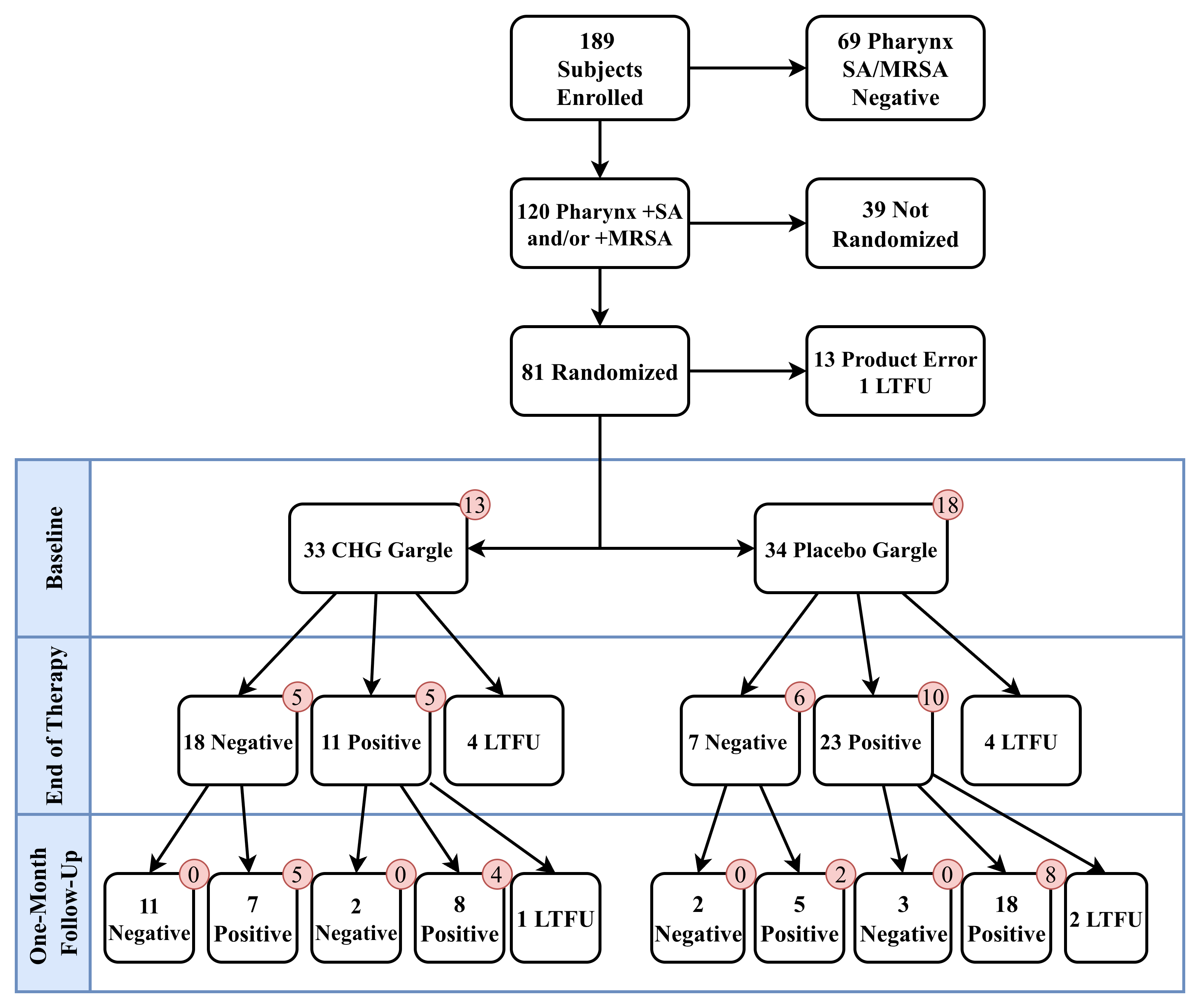

Supplement: ofag072_Supplementary_Data [file ofag072_supplementary_data.zip › Supplementary Figure 1.png]

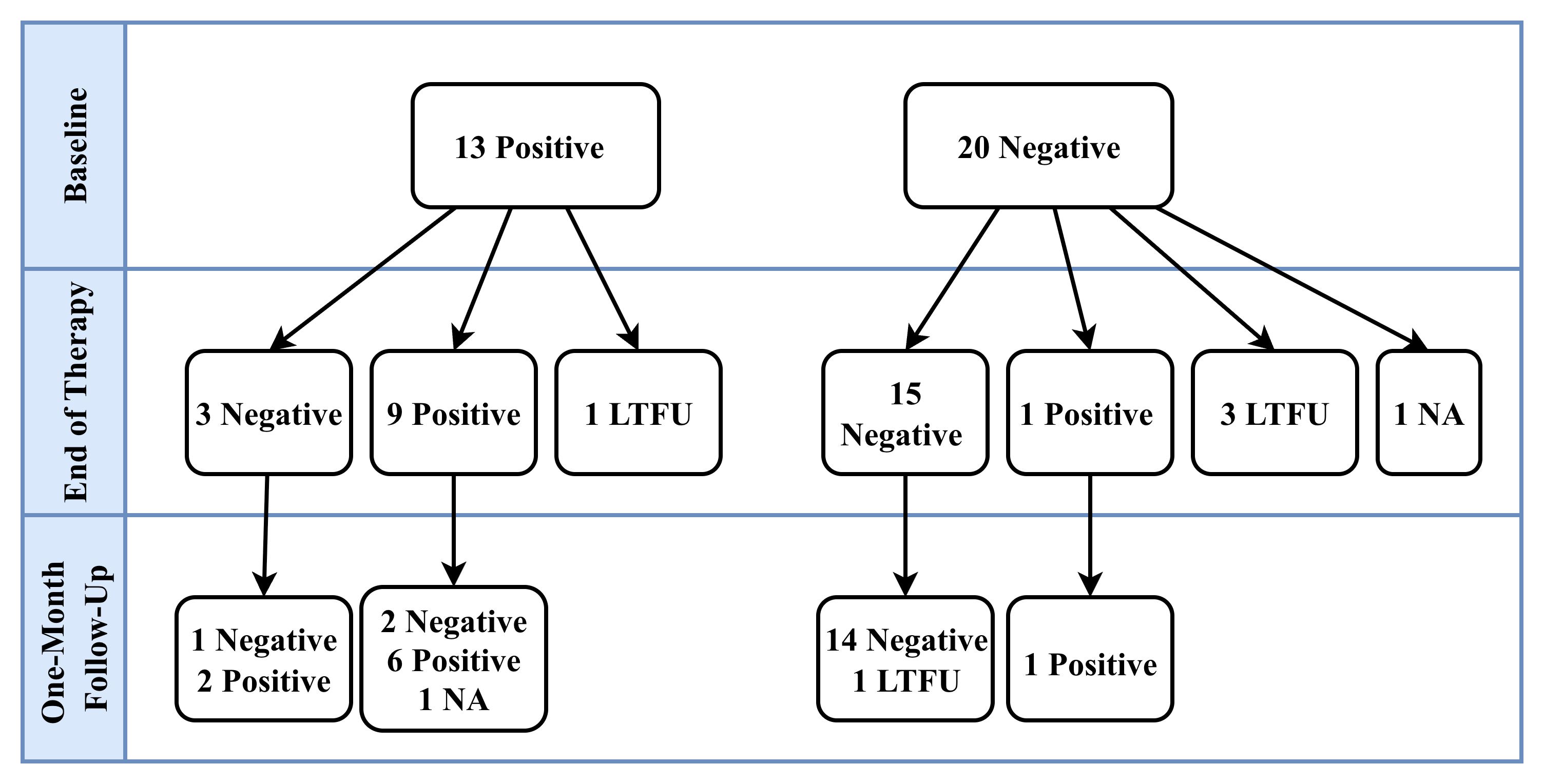

Supplement: ofag072_Supplementary_Data [file ofag072_supplementary_data.zip › Supplementary Figure 2.png]

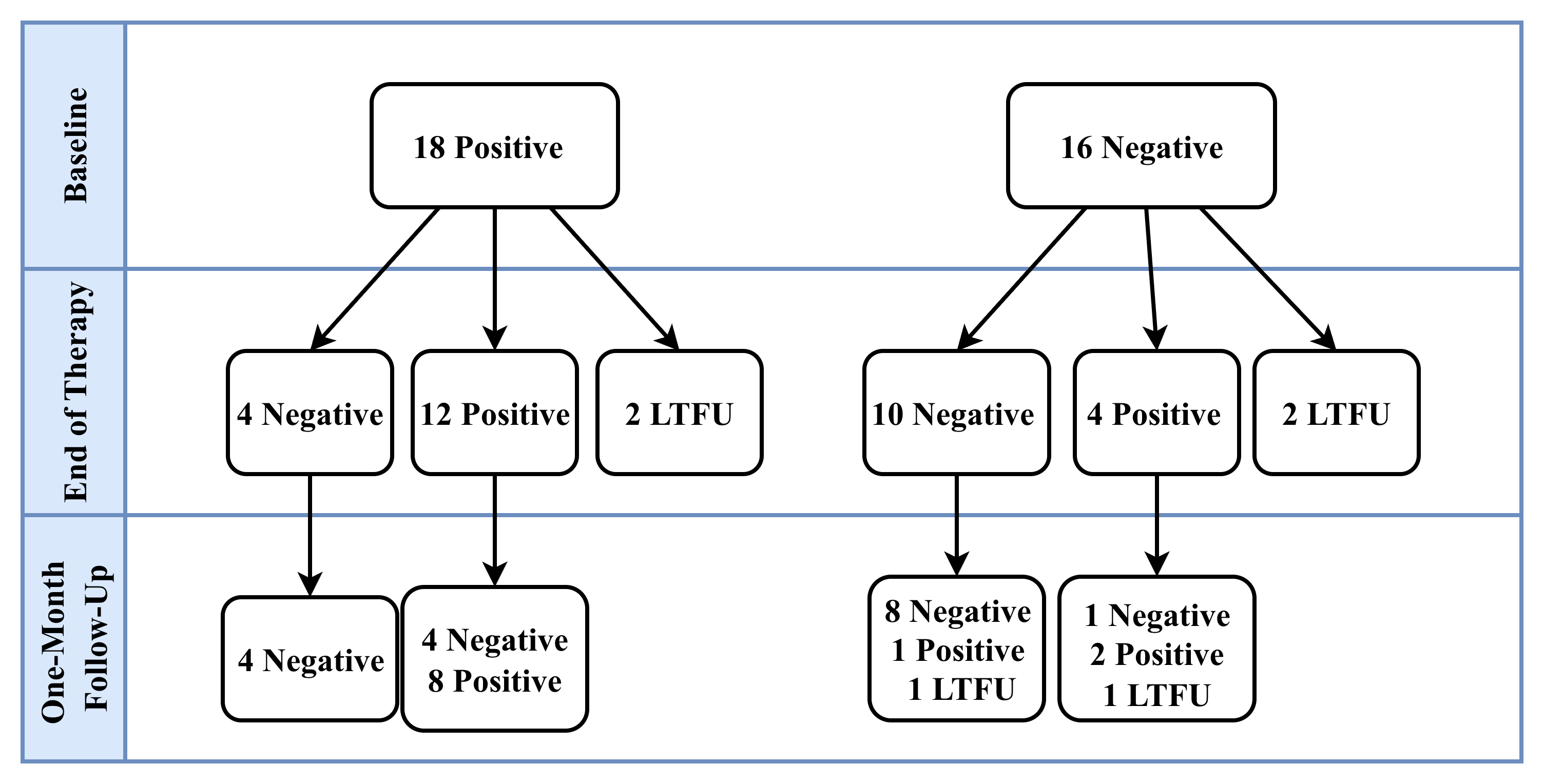

Supplement: ofag072_Supplementary_Data [file ofag072_supplementary_data.zip › Supplementary Figure 3.png]
